# High Throughput Isolation and Data Independent Acquisition Mass Spectrometry (DIA-MS) of Urinary Extracellular Vesicles to Improve Prostate Cancer Diagnosis

**DOI:** 10.3390/molecules27238155

**Published:** 2022-11-23

**Authors:** Hao Zhang, Gui-Yuan Zhang, Wei-Chao Su, Ya-Ting Chen, Yu-Feng Liu, Dong Wei, Yan-Xi Zhang, Qiu-Yi Tang, Yu-Xiang Liu, Shi-Zhi Wang, Wen-Chao Li, Anke Wesselius, Maurice P. Zeegers, Zi-Yu Zhang, Yan-Hong Gu, W. Andy Tao, Evan Yi-Wen Yu

**Affiliations:** 1 State Key Laboratory of Bioelectronics, National Demonstration Center for Experimental Biomedical Engineering Education, Southeast University, Nanjing 210096, China; 2 EVLiXiR Biotech, Nanjing 210032, China; 3 Department of Colorectal Tumor Surgery, The First Affiliated Hospital of Xiamen University, School of Medicine, Xiamen University, Xiamen 361003, China; 4 Department of Mental Health Research, Xiamen Xianyue Hospital, Xiamen 361012, China; 5 Key Laboratory of Environmental Medicine and Engineering of Ministry of Education, Department of Epidemiology & Biostatistics, School of Public Health, Southeast University, Nanjing 210009, China; 6 Bell Mountain Molecular MedTech Institute, Nanjing 210032, China; 7 Medical School of Southeast University, Nanjing 210009, China; 8 Key Laboratory of Environmental Medicine Engineering, Ministry of Education, School of Public Health, Southeast University, Nanjing 210009, China; 9 Department of Urology, Affiliated Zhongda Hospital of Southeast University, Nanjing 210009, China; 10 Department of Epidemiology, CAPHRI Care and Public Health Research Institute, Maastricht University, 6229ER Maastricht, The Netherlands; 11 School of Nutrition and Translational Research in Metabolism, Maastricht University, 6229ER Maastricht, The Netherlands; 12 Department of Pathology, Jiangxi Maternal & Child Health Hospital, Nanchang 330006, China; 13 Department of Oncology, The First Affiliated Hospital of Nanjing Medical University, Jiangsu Province Hospital, Nanjing 210029, China; 14 Departments of Chemistry and Biochemistry, Purdue University, West Lafayette, IN 47907, USA

**Keywords:** prostate cancer, extracellular vesicles, liquid biopsy, diagnostic biomarker

## Abstract

Proteomic profiling of extracellular vesicles (EVs) represents a promising approach for early detection and therapeutic monitoring of diseases such as cancer. The focus of this study was to apply robust EV isolation and subsequent data-independent acquisition mass spectrometry (DIA-MS) for urinary EV proteomics of prostate cancer and prostate inflammation patients. Urinary EVs were isolated by functionalized magnetic beads through chemical affinity on an automatic station, and EV proteins were analyzed by integrating three library-base analyses (Direct-DIA, GPF-DIA, and Fractionated DDA-base DIA) to improve the coverage and quantitation. We assessed the levels of urinary EV-associated proteins based on 40 samples consisting of 20 cases and 20 controls, where 18 EV proteins were identified to be differentiated in prostate cancer outcome, of which three (i.e., SERPINA3, LRG1, and SCGB3A1) were shown to be consistently upregulated. We also observed 6 out of the 18 (33%) EV proteins that had been developed as drug targets, while some of them showed protein-protein interactions. Moreover, the potential mechanistic pathways of 18 significantly different EV proteins were enriched in metabolic, immune, and inflammatory activities. These results showed consistency in an independent cohort with 20 participants. Using a random forest algorithm for classification assessment, including the identified EV proteins, we found that SERPINA3, LRG1, or SCGB3A1 add predictable value in addition to age, prostate size, body mass index (BMI), and prostate-specific antigen (PSA). In summary, the current study demonstrates a translational workflow to identify EV proteins as molecular markers to improve the clinical diagnosis of prostate cancer.

## 1. Introduction

The incidence of prostate cancer has experienced an unprecedented increase in diagnosis over the past few decades [1], which made it to be the most common malignancy among men in the United States, accounting for 25.6% of all cancer diagnoses, with a mortality rate of 13.7% [2]. Accurate diagnosis of prostate cancer has an important clinical impact on cancer therapy and overall survival. Currently, screening for prostate cancer begins with a test that measures the amount of prostate-specific antigen (PSA); however, PSA usefulness in prostate cancer screening has been widely argued since an elevated PSA level may be caused by prostate cancer but can also by other conditions, including an enlarged prostate (benign prostatic hyperplasia) and inflammation of the prostate (prostatitis), which may cause “false-positive” results [3]. In recent years, several new biomarkers supplementing the role of PSA have become available for the early onset of prostate cancer. As one of the promising fluid biopsies in the human body, urine may be enriched with prostate cancer biomarkers, which include prostate cancer cells, DNAs, RNAs, proteins, exosomes, and metabolites. Among them, extracellular vesicles (EVs) have drawn increasing attention. EVs, which are classified into exosomes (30–100 nm in diameter) and microvesicles (50–1000 nm in diameter), contain DNA, RNA, and proteins encapsulated into the bilayer lipid membranes [4]. An EV’s molecular cargo may mirror the content of donor cells, such as cancer cells, which makes it a valuable source of tumor-related biomarkers.

EVs contain a restricted set of proteins, microRNA, mRNA, and DNA and play important roles in cell-cell communication by transferring their content to target cells [5]. According to Chen et al. [6], EVs are robustly produced by cancer cells and markedly affect the primary tumor microenvironment (TME), including the immune ecosystem as well as distant metastatic niches, further facilitating tumor growth and metastasis. Thereby, accumulative studies suggest that EVs found in biofluids, such as urine, can be a promising source for disease diagnosis [7,8,9]. However, there are certain challenges to defining and standardizing reliable methods for EV isolation for clinical utility, as currently, multiple methods, including differential centrifugation, density gradient centrifugation, size-exclusion chromatography (SEC), and/or affinity chromatography, microfluidic devices, and synthetic polymer-based precipitation reagents, are used [10]. However, for practical clinical applications, how to extract EVs with high throughput and reproducibility is an urgent challenge to be addressed. To solve this issue, in recent years, our group has developed an automatic extraction device [11] to complement our previously developed magnetic bead EVTRAP [12]. The device, named as EVrich, uses the principle of magnetic separation to distribute and elute EVs captured by magnetic beads, which allows us to extract EVs from multiple samples in parallel.

In addition, recent advances in mass spectrometry (MS) allow for the detection and quantification of thousands of proteins in cells, tissues, and biological fluids. As a powerful analytical tool, MS has been used to unravel the fundamental role of EVs in cancer, e.g., the modulation of the tumor microenvironment, mediating immune evasion, and contributing to metastasis [13,14]. MS-based proteomics is also cogent for exploring EVs as a source of diagnostic markers, prognostic markers (severity of the outcome), and predictive markers (e.g., response to treatment). Data-dependent acquisition (DDA) method and data-independent acquisition (DIA) are two commonly used acquisition modes in MS. DIA is a powerful tool for disease biomarker discovery because of its improved coverage, sensitivity, and reproducibility compared to DDA methods [15]. Different library-based search strategies for DIA data have been reported. The method of fractionated DDA-DIA is to build the spectral library through DDA after sample fractionation. It performs better than the DDA, and good quantitative results can be obtained without relying on long gradients. The disadvantage is that this method can be very time-consuming when only a small number of samples are tested [16]. Gas-phase fractionation (GPF)-DIA is a method for obtaining, queuing, and validating DIA data with only a DIA chromatographic library using gas-phase fractionation, the advantage of which is experiment-specific, is always up-to-date and does not require offline fractionation for library generation [15,17]. On the other hand, direct DIA technology can achieve a larger dynamic range, higher recognition reproducibility, and higher quantitative sensitivity and accuracy than DDA technology without additional library construction [18,19]. In this study, we integrate these three DIA methods to analyze 40 clinical samples collected from prostate disease patients so as to obtain more comprehensive and credible quantitative results. In this study, we applied the high throughput, automatic platform, EVrich, for efficient isolation of EVs, and then the integrated DIA-MS strategy to subsequently analyze the samples. The study presents proteomic signatures that are valuable in facilitating the screening and diagnosis of prostate cancer cases.

## 2. Results

### 2.1. Automated Isolation and Characterization of Urinary EVs

**Figure 1:**
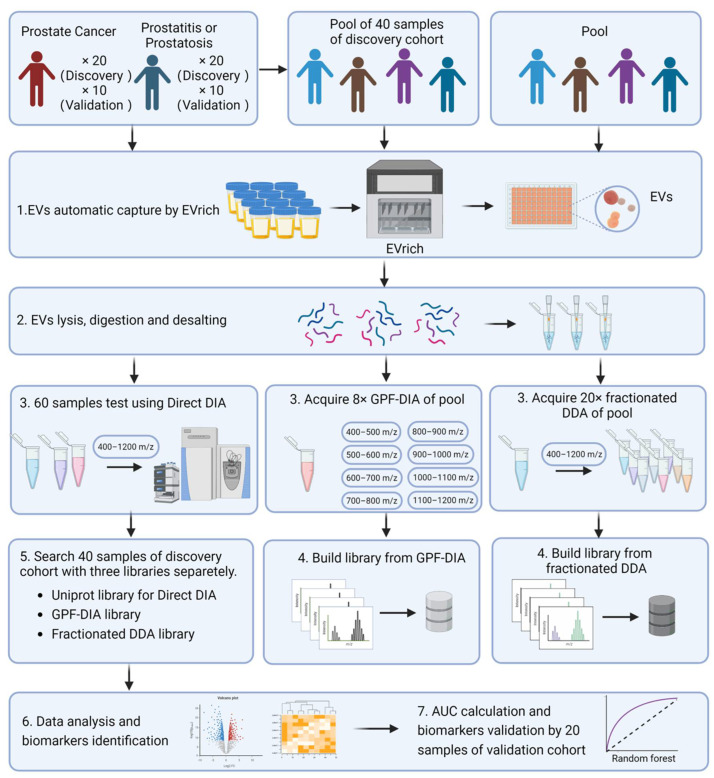
Experimental Design and Workflow of the Study. (Created by BioRender.com) EVs were separated in urine samples collected from all participants. We then used the EVTRAP method to capture and isolate the EVs. For the establishment of the library for MS searching, in this step, the samples from all 40 participants of the discovery cohort were pooled as the main library for GPF-DIA-base search, while other independent 40 biological samples were pooled and conducted as a secondary library for fractionated DDA-base DIA search. Meanwhile, the particle number and size of EVs were examined using Nanoparticle tracking analysis (NTA). In addition, the size and structure of extracted EV were examined by morphology using Transmission electron microscopy (TEM). Subsequently, the isolated EVs were lysed, digested, and desalted into peptides, then three strategies were performed for MS measurement and searching separately. The individual EV proteins were analyzed for discrimination between prostate cancer cases and non-prostate cancer controls, with the annotation on potential mechanistic pathways. Lastly, a random forest model for accessing the performance of classifying prostate outcomes was performed.

**Figure 2:**
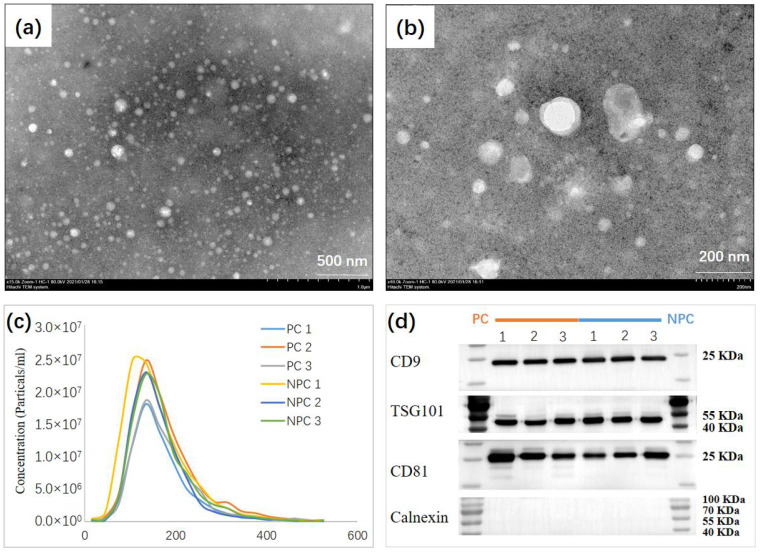
Biochemical and Morphological Characterization of EVs. (**a**,**b**), TEM image of urine EV from selected individuals showing the typical exosome-round size (~150 nm) and morphology. (**c**) NTA of urine EV revealing a similar EV mode (~150 nm) between prostate cancer cases (n = 3), and non-prostate cancer controls (n = 3). (**d**) Representative immunoblots indicating that the EV isolated from the urine of selected individuals present enrichment of the EV markers CD9, TSG101, and CD81 but do not contain Calnexin used as control.

The experimental design and workflow are depicted in detail in Figure 1. EVs were separated in urine samples collected from all participants. In total, 60 participants were recruited in the current study, including 40 participants as the discovery cohort (20 prostate cases and 20 non-prostate cancer controls) and 20 participants as the independent validation cohort (10 prostate cancer cases and 10 non-prostate cancer controls). A difference in PSA (*p* = 0.006) was observed upon prostate cancer outcome (i.e., cases vs. controls), which the prostate cancer cases indicated a higher PSA (25.98 ± 20.77 ng/mL), while a lower PSA (8.28 ± 3.61 ng/mL) was observed in non-prostate cancer controls. However, no difference was found in terms of age (*p* = 0.275), body mass index (BMI; *p* = 0.534), and prostate size (*p* = 0.407) (Supplementary Table S1). For the isolation process, we used EVTRAP beads corresponding to automated magnetic bead separation EVrich [11,20] to capture and isolate the EVs for 40 samples in the discovery cohort first. A batch of 1 mL urine samples was mixed with EVTRAP beads and loaded onto the first well for 30 min of incubation, and washed with the provided wash buffer once. The beads were separated from urine and washed twice in two separate wells before elution by triethylamine in the last two wells. Purified EVs were dried under vacuum and stored at −80 °C until used. For the characterization of the EVs isolated by EVrich, three samples were randomly selected from each group. The size and structure of extracted EV were examined by Transmission electron microscopy (TEM). These nanovesicles showed the typical exosome-like round morphology by TEM (Figure 2a,b). Particle number and size of EVs were examined using Nanoparticle tracking analysis (NTA). Analysis of the urine EV size (mode) by NTA revealed a slight decrease in the EV mode size in prostate cancer cases vs. non-prostate cancer controls, although no significant differences were observed. The average mode size of urine EV was 150 nm in all groups (Figure 2c). In addition, the presence of the common EV markers TSG101, CD9, and CD81, and the absence of negative control Calnexin, an endoplasmic reticulum protein, were validated by performing western blot (WB) analyses (Figure 2d). The WB results indicate that overall EV isolation by EVTRAP based on the EVrich platform is reproducible and specific.

### 2.2. Quantitative Measurement of Urinary EV Proteins through Integrated DIA-MS

First, the isolated EVs were lysed, digested, and desalted into peptides, then three strategies were performed for MS measurement and searching separately; (1) a direct searching [18] based on Uniprot library was conducted, without a requirement of spectral libraries to analyze DIA data, which was denoted as Direct-DIA. Here we cyclically fragmented all peptides across the entire precursor mass range of 400–1200 m/z; (2) a gas-phase fractionated (GPF) library-based searching was denoted as GPF-DIA. In this case, we generated the GPF-DIA spectral library using the pooled sample processed similarly to the individual samples. The pooled sample was injected into the MS eight times representing different window placements, i.e., 400–500 m/z, 500–600 m/z, 600–700 m/z, 700–800 m/z, 800–900 m/z, 900–1000 m/z, 1000–1100 m/z, and 1100–1200 m/z; (3) A fractional DDA based searching was denoted as DDA-DIA. For fractionated DDA sample preparation, 40 biological samples were mixed to form a pooled sample, and EVs were isolated through EVTRAP. After lysis, enzymatic hydrolysis, and salt removal, the isolated EVs were fractionated by HPLC, and a total of 20 fractions were obtained for DDA detection. The results of DDA were used to build the library for the DIA search consisting of 6104 proteins and 40,955 peptides, as well as for the GPF search consisting of 3535 proteins and 28,522 peptides (Supplementary Table S1). Overall, 2969 EV proteins (30,782 EV peptides) for Direct-DIA, 3282 EV proteins (27,668 EV peptides) for GPF-DIA, and 3273 EV proteins (22,690 EV peptides) were obtained (Supplementary Table S2 and Supplementary Figure S1a). Though the process of DDA-DIA was more complicated and time-consuming compared to the other two strategies (i.e., Direct-DIA and GPF-DIA), a larger proteome could be gained with 388 detected additionally, of which seven were uniquely identified in this method with significantly differentiated between prostate cancer outcomes. In addition, the GPF-DIA method also detected 282 additional EV proteins, of which eight differentiated proteins were uniquely identified (Supplementary Figure S1a). Therefore, the integration of DDA-DIA, GPF-DIA, and Direct-DIA increased the number of EV proteins and further enhanced the capacity of EV-based biomarker identification. To ensure the discovery cohort (n = 40) free from included participants per se that might confound analysis, we independently collected 20 participants as a validation cohort for comparison to the discovery cohort and as a test dataset for further establishment of the classification model based on a random forest algorithm. We then characterized their urine EV proteome based on the same protocol as Direct-DIA. Through this, we observed a 2376 out of 2923 (81%) urine EV proteins could be replicated with comparison to the discovery cohort (2969 urinary EV proteins based on Direct-DIA) (Supplementary Table S2 and Supplementary Figure S1b), which indicated the majority of urinary EV proteins are unlikely to be detected by chance. For assessing the reproducibility between the discovery cohort and the validation cohort for those EV proteins overlapped in different DIA search strategies, we calculated the Spearman correlation coefficients and found the mean values were 0.94 and 0.97 upon prostate cancer cases and non-prostate cancer controls separately, which indicated a high reproducibility of the current EV proteomics workflow. In addition, by calculating the range of EV protein abundances for each participant, we found that except for a few participants, the mean abundance was mostly located within an exponent (i.e., ×10) regardless of DIA strategies and cohorts, which suggests high stability of the measurement protocol (Supplementary Figure S1c–f).

### 2.3. EV Protein Profiling and Comparative Analyses for Prostate Cancer Outcome

**Figure 3:**
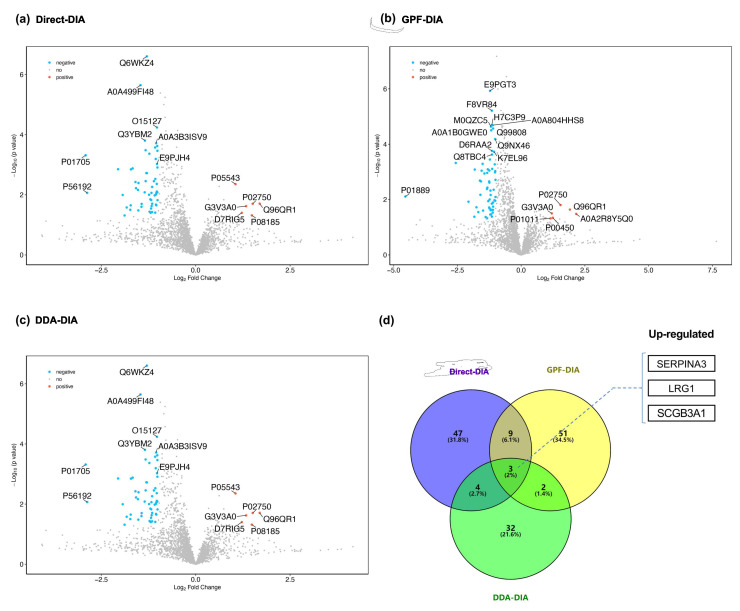
Comparative Analysis of EV Proteins between Prostate Cancer Cases and Non-prostate Cancer Controls. To distinguish the EV protein characteristics between prostate cancer cases and non-prostate cancer controls, we compared and identified the upregulated or downregulated EV proteins, then summarized the overlapped EV proteins across three search strategies. (**a**–**c**) Volcano plots (Direct-DIA, GPF-DIA, and DDA-DIA, respectively) for statistical differences between the two groups (prostate cancer cases vs. non-prostate cancer controls) were assessed with the Mann-Whitney test. (**d**) Venn diagram for assessing the overlap of differentiated EV proteins across three search strategies.

The results of significantly differentiated EV proteins in prostate cancer cases compared with non-prostate cancer controls based on different methods (i.e., Direct-DIA, GPF-DIA, and DDA-DIA) are summarized in the volcano plot shown in Figure 3a–c, and most prominent proteins are indicated. Among the upregulated proteins, five were highly enriched in EVs driven from the urine of cases with prostate cancer, while the other 13 EV proteins showed downregulated compared prostate cancer cases to non-prostate cancer controls.

**Figure 4:**
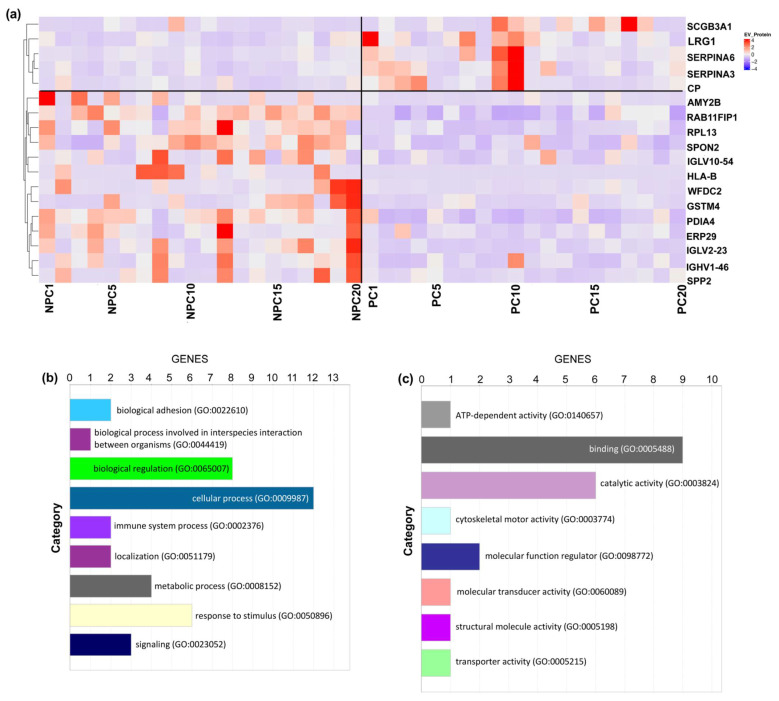
Unsupervised Clustering and Biological Annotation for Differentiated EV Proteins. (**a**) Unsupervised clustering of all included participants using the 18 EV proteins with a significant difference. Each column indicates one participant, and each row indicates one EV protein. The colors indicate either upregulated (red) or downregulated (purple). (**b**) Biological process analysis. (**c**) Enriched pathway analysis.

For further evaluation of the expression of identified 18 EV proteins in prostate cancer outcomes, we performed an unsupervised clustering of the entire cohort of cases and controls, which showed a distinct separation between prostate cancer cases and non-prostate cancer controls (Figure 4a). The clustering yielded a clear pattern of the five upregulated EV proteins (i.e., SERPINA3, SCGB3A1, LRG1, SERPINA6, and CP) enriched in the prostate cancer cases.

We then submitted the dataset of 18 significantly differentiated EV proteins to Gene Ontology (GO) Database for annotation, visualization, and integrated discovery based on the PANTHER platform [21]. The biological process analysis of the significantly differentiated EV proteins was enriched in metabolic process, biological regulation, response to stimulus, immune system process, and localization (Figure 4b). In addition, enriched pathway analysis included the cadherin signaling pathway, cytoskeletal regulation by Rho GTPase, inflammation signaling pathway, integrin signaling pathway, and nicotinic acetylcholine receptor signaling pathway, which were related to cancer development (Figure 4c). However, due to the limitation of database-based annotation, the mechanistic pathways warrant further study.

Among the identified differentiated EV proteins, three EV proteins (i.e., Alpha-1-antichymotrypsin (SERPINA3), Leucine-rich alpha-2-glycoprotein (LRG1), and Secretoglobin family 3A member 1 (SCGB3A1)) were found to be consistently upregulated in all methods (Figure 3d).

SERPINA3 has been reported to principally works as an inhibitor in maintaining cellular homeostasis; it is a matricellular acute-phase glycoprotein that appears to be the sole nuclear-binding secretory serpin [22]. Several studies have emerged in recent years demonstrating its link to cancer biology [23,24,25,26]. As a typical acute-phase protein, SERPINA3 is regulated by inflammatory cytokines so that its expression is increased in the inflammatory response; overexpression of SERPINA3 predicts that damage tends to occur in the body contributing to decreased cell adhesion ability and inhibition of apoptosis. It is revealed that the up-regulation of SERPINA3 is positively associated with malignant tumors [27,28].

LRG1 has previously been implicated as a promising biomarker for aggressive prostate cancer in an animal model [18]. Several proteomic studies based on serum samples have found LRG1 to be elevated in more aggressive prostate cancer [29,30]. Though emerging studies have reported LRG1 associated with unfavorable outcomes in multiple cancer types, including prostate cancer, the function of LRG1 is so far largely unknown. While the involvement of TGFb signaling regulates angiogenesis and invasion, LRG1 is a key regulator of myeloid differentiation, expressed in most myeloid lineages [31] and induced in response to specific acute-phase cytokines [32]. These implicate a significant biological role of LRG1 in inflammation but also several important hallmarks of cancer development and progression, which might shed light on the highly diverse outcomes among patients diagnosed with high-risk and metastatic prostate cancer.

Though there is no study yet to investigate the relationship between SCGB3A1 and prostate cancer, it has been found to be upregulated in breast cancer [33] and testicular cancer [33]. This gene was initially characterized based on its differential expression between malignant and normal tissues and assumed in relation to methylation. Both the initial study of SCGB3A1 and subsequent studies have documented a strong association between promoter hypermethylation; however, the exact function of the protein remains unknown.

To reduce the influence of the clinical background on the identification of the urinary EV proteins related to prostate cancer outcome, we performed a logistic regression analysis with adjustments of age, BMI, prostate size, and PSA, where the results indicated SERPINA3, LRG1, and SCGB3A1 (upregulated EV proteins) to be positively associated with the risk of prostate cancer (OR 1.19, 95% CI 1.05, 1.33; OR 1.10, 95% CI 1.03, 1.17; OR 1.08, 95%CI 1.02, 1.14, respectively). (Supplementary Figure S2a). Furthermore, to assess the independent effect of the three EV proteins identified above with PSA, we presented a scatter plot to show whether there is a concurrent change between each EV protein and PSA, in which no clear association pattern could be observed, indicating the possibility of those biomarkers interacted by PSA are minimal. Therefore, the current study provides new assumptions that EV proteins may be involved in other mechanistic pathways distinct from the conventional biomarker (i.e., PSA [34]), which might address the limitation of PSA in prostate cancer diagnosis (Supplementary Figure S2b–d).

**Figure 5:**
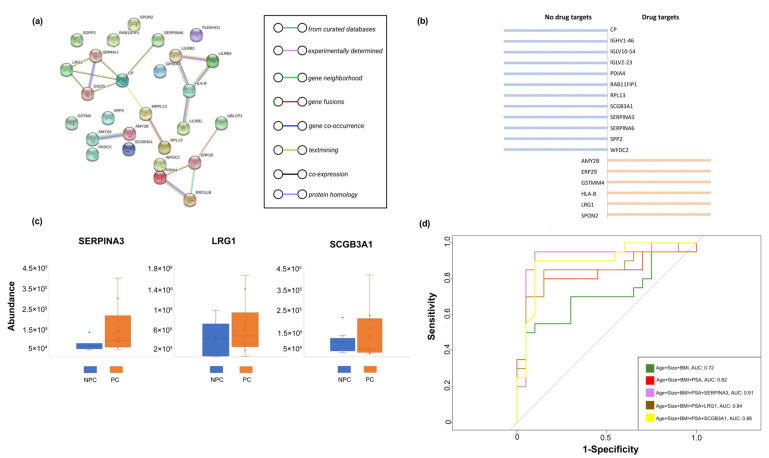
Post analyses for the Differentiated Urinary EV Proteins. (**a**) Results of the STRING interaction analysis (PPI enrichment); (**b**) Established druggable targets from the identified EV proteins. (**c**) Comparison of consistently upregulated EV proteins between prostate cancer cases and non-prostate cancer controls based on the validation cohort. (**d**) ROC curves and the corresponding AUCs for the components of clinical information and identified EV proteins.

The STRING interaction analysis (Figure 5a) revealed that the components of the EV proteomic signature detected in the urine of prostate cancer cases were enriched in their interactions (PPI enrichment, *p* = 0.032) with the highest confidence (0.9), forming four potential PPI modules consisting of 17 nodes and 18 edges: SERPINA3-LRG1-GIG25-CP-MRPL13-PRL3, LILRB2-LILRB4-HLA-B-LILRB1, PDIA4-ERO1LB-ERP29-UBLCP1, and AMY2A-AMY2B. In addition, we found six (33%) EV proteins that had been developed as drug targets based on the DrugBank database (v5.1.8), while others (67%) remained unavailable and deserved further development (Figure 5b).

### 2.4. Analysis of Identified EV Proteins in Classification of Prostate Cancer Cases and Controls

To validate our results, we additionally recruited 20 participants (i.e., 10 prostate cancer cases and 10 non-prostate cancer controls) with the same protocols for sample collection and EVs measurement. In comparison to the discovery cohort, the identified EV proteins (i.e., SERPINA3, LRG1, and SCGB3A1) gained from the validation cohort clearly complied with the discovery cohort, which indicated the effect of the identified EV proteins on prostate cancer were stable (Figure 5c).

In addition, we first examined the fitted model with multiple combinations of clinical information and identified EV proteins in the discovery cohort (i.e., training dataset), while the validation cohort (i.e., test dataset) was used to make a classification based on the established model obtained from the discovery cohort. We then constructed a receiver operating characteristic curve (ROC) based on a random forest algorithm and calculated the area under it (i.e., the AUC), where the results showed SERPINA3, LRG1, and SCGB3A1 added classified value (added AUC; 0.09, 0.02, 0.04, respectively) in addition to age, prostate size, BMI, and PSA, suggesting the clinical value by incorporating EV biomarkers in prostate cancer diagnosis (Figure 5d).

## 3. Discussion

In the current study, we applied chemical affinity-based magnetic beads, EVTRAP, and a parallel magnetic bead separation station, EVrich, to isolate urinary EVs, followed by profiling EV proteomes to differentiate prostate cancer cases from prostatitis or prostatosis (non-prostate cancer controls). We observed several EV proteins, particularly SERPINA3, LRG1, and SCGB3A1, which showed a consistent difference between prostate cancer cases and non-prostate cancer controls across three DIA methods, which could be suggested as potential biomarkers and deserve further verification.

Although we integrated three DIA-based methods that showed great consistency given that the majority of EV proteins could be replicated, each DIA method also contributed to unique sets of proteins. When compared with Direct-DIA, DDA fractionation library search and GPF-based library search contributed to providing additional differentiated EV proteins, indicating that single library-based analysis would still be considered to cause some information loss. Therefore, a more comprehensive search strategy with multiple libraries is relatively more cogent and should be recommended.

EVs have been found to play a role in numerous biological processes, including sending inhibitory or stimulatory growth signals to close and distant cell human sites. EVs can affect the extracellular matrix (ECM) by modulating tumor immune responses and can even transfer active oncogenes, e.g., epidermal growth factor receptor (EGFR) or the mutant form of Kirsten rat sarcoma virus (KRAS) [35,36,37,38]. Thus, EVs represent a rich source of potential biomarkers that could contribute to the diagnosis of cancer and to the prognosis of outcomes (prognostic markers) and responses to treatment (predictive markers). Moreover, EVs have been found in various bodily fluids, including blood, saliva, and urine [39]. Therefore, the analysis of the diagnostic function, molecular composition, and biological mechanisms of EVs is relevant to the field of liquid biopsy of interest in translating into non-invasive clinical practice.

To the extent of our knowledge, this is the first description of urinary EV proteome in prostate cancer cases compared with non-prostate cancer controls but with benign prostatic hyperplasia or inflammation of the prostate (i.e., prostatitis or prostatosis). Given that the symptoms of prostate cancer are similar to prostatitis or prostatosis [40], it leads to increasing difficulty in diagnosis. According to Litwin [41], invasive clinical examinations (i.e., aspiration examination for tissue biopsy) enhance the accuracy of prostate cancer diagnosis; however, due to its inherent features that may cause health burden, extra biomarkers with valid performance in the early stage of the clinical pathway are needed. Furthermore, there is no previously reported evidence describing the classifying of different diagnoses regarding prostate cancer and prostatitis or prostatosis in addition to the conventional method, i.e., PSA, while in the current study, there is an enhanced performance when adding identified EV proteins to the conventional model with age, BMI, prostate size, and PSA. We expect the integration of molecular markers such as those from EVs with clinical parameters will be a future direction for better diagnosis and precision medicine.

The gene ontology enrichment and molecular function analysis suggested EV proteins differentiated in prostate outcome were related to signaling and binding, catalytic activity, cellular process, transportation, and immune/inflammatory responses. These findings indicated a role for these EV proteins in biological regulation and in the cancer signaling pathway. Alternatively, the categories of these differentiated EV proteins could reflect a role in the (EV-mediated) response to prostate damage in general, particularly for those consistently upregulated EV proteins, which were associated with extracellular matrix organization and response to carcinogenesis.

Several limitations drew our attention. This is a study to demonstrate a translational platform for biomarker discovery. Rigid biomarker discovery requires a much larger sample size and collection that provides sufficient power to assess differential expression in prostate cancer cases and non-prostate cancer controls. Despite the strategy of using three different DIA methods to enhance the consistency of the identified EV proteins, the low overlap of differentiated EV proteins across methods should be noted. With the consideration of sample size, multiple testing was not applied in the current study, which may have resulted in potential false-positive findings. While we identified some indications of EV proteins, our findings warrant further investigations to confirm the biomarkers and their interactions in the context of prostate cancer development. In addition, while high-throughput techniques offer a high-resolution view of the relevant molecules related to the prostate, we cannot rule out the presence of additional, low-abundance molecules that could not be detected from the depth of the EV proteome generated.

## 4. Materials and Methods

### 4.1. Materials and Reagents

EVTRAP beads were purchased from Tymora Analytical Operations (West Lafayette, IN, USA). Antibodies used in western blotting included CD9 (13403S, CST, MA, USA), CD81 (ab79559, Abcam, MA, USA), TSG101 (ab125011, Abcam, MA, USA), and Calnexin (ab133615, Abcam, MA, USA). EVs lysis reagents, including sodium deoxycholate, sodium lauroylsarcosinate, Tris (2-carboxyethyl) phosphine, and 2-Chloroacetamide were all obtained from Millipore-Sigma, MO, USA. Other reagents are common laboratory reagents without specific supplier requirements.

### 4.2. Participants Recruitment and Demographic Characteristics Assessment

All participants were informed about the study’s purpose during the admission interview, with written consent prior to enrolling in the study. The study design and conduct complied with all relevant regulations regarding the use of human participants and was in accordance with the criteria set by the Declaration of Helsinki. The study’s protocol was approved by the Ethics Committee of the First Affiliated Hospital of Nanjing Medical University (2021-SR-167, 7 April 2021), Nanjing, China.

The participants were recruited for this study at a tertiary-care hospital in Nanjing, China. All participants were local residents and of Han ancestry. The medical records of each participant were reviewed by trained doctors or nurses, and clinicopathological characteristics of prostate cancers at the diagnosis were prospectively gathered on dedicated case report forms. None of the participants were undergoing other medical interventions. The diagnosis of prostate cancer was confirmed with histology [42]. A urine sample (20 mL) was taken at the time when the participant entered the study but before going to surgery or chemotherapy, and stored in sterilized and portable plastic containers, then transferred to a −80 °C facility within 4 h after collection. In total, 60 participants were included in the current study, including 40 participants as the discovery cohort (i.e., 20 prostate cancer cases and 20 non-prostate cancer controls) and 20 participants as the validation cohort (i.e., 10 prostate cancer cases and 10 non-prostate cancer controls).

### 4.3. EVs Automatic Isolation by EVrich

The EVrich system was used as described previously [11]. Firstly, the magnetic beads and urine were added to the predetermined 96-well plate according to the ratio of 20 μL: 1 mL and mixed for half an hour, by which magnetic beads had completed EVs capture. The magnetic beads were then moved to three holes for three steps of washing. Finally, EVs were obtained after two rounds of elution. The eluted EVs were collected, combined, and dried in a vacuum centrifuge.

### 4.4. Characterization of EVs by Transmission Electron Microscopy (TEM)

The EV-TEM solution was prepared by dispersing the EVs corresponding to 1 mL urine sample in 200 μL PBS. Then 10 μL of EV-TEM solution was dropped into a 200-mesh Formvar carbon-coated copper grid. After naturally drying, the sample was incubated with 2% phosphotungstic acid solution for at least 2 min at 25 °C for negative staining. TEM images of electric vehicles were performed on a Hitachi H-8100 electron microscope (Hitachi, Tokyo, Japan).

### 4.5. Nanoparticle Tracking Analysis

EVs, isolated from 1 mL of the urine sample by EVrich, were firstly dried to a powder and diluted in 0.5 mL with PBS for further NTA analysis. The size of EVs was determined using a ZetaView (Particle Metrix, Meerbusch, Germany) according to the standard protocol. Calibration was first performed with 100 nm polystyrene particles diluted 250,000 times with purified water. The minimum brightness was set to 20, and the sensitivity and shutter were set to 70 and 100, respectively.

### 4.6. Western Blotting Analysis

EVs corresponding to 1 mL urine sample was boiled with 20 µL LDS loading buffer for 5 min to obtain protein lysates. Then, these protein lysates were loaded into a 15% SDS-PAGE under voltage 190 V for 70 min. Further, the samples in the PAGE were transferred into PVDF membranes. After being blocked with 1% BSA, the membranes were incubated with CD9, CD81, TSG101, and Calnexin antibodies at 25 °C for 4 h. Finally, secondary antibodies were incubated with the membranes for further scanned by chemiluminescence imager (ImageQuant LAS500) to achieve the immunoassay and quantitation.

### 4.7. Preparation of EV Samples for LC-MS

The eluted EVs were added into 100 μL PTS lysate and then boiled in a 95 °C water bath for 10 min. Then, it was cooled to ambient temperature and diluted five-fold with 50 mM TEAB buffer. A BCA assay determined total protein content, and then Lys-C enzyme (EVLiXiR) was added according to the remaining sample protein content: enzyme = 100:1 and incubated at 37 °C for 3 h. The trypsin was added according to the sample protein content: trypsin = 50:1 and incubated overnight in a 37 °C water bath to further digest the peptides. An amount of 50 μL of 10% TFA was added to acidify the sample to terminate the enzymatic digestion, and five times the volume of ethyl acetate was added and vortexed for 2 min and centrifuged at 15,000× *g* for 3 min. The upper layer of ethyl acetate was removed, and the lower phase was lyophilized in a refrigerated vacuum centrifuge. After desalting by using an 8 mm Capture Disk (3 M Empore 2240-SDB-XC) according to the manufacturer’s instructions, all samples were freeze-dried in a refrigerated vacuum centrifuge and stored at −80 °C.

### 4.8. LC−MS/MS Analysis

The desalted samples were re-solubilized using 0.1% formic acid, and appropriate amounts of peptides were taken from each case for chromatographic separation using a nanoliter flow rate Easy-nLC 1200 chromatography system (Thermo Scientific, Waltham, MA, USA). The sample was separated on a 25 cm in-house packed column (360 µm OD × 75 µm ID) containing C18 resin (2.2 µm, 100 Å; Michrom Bioresources, CA, USA) at a flow rate of 300 nL/min. Solution A was 0.1% formic acid in water, and solution B was 80% ACN/0.1% formic acid. The chromatographic column was equilibrated with 100% of liquid A, and 0–3 min is 2–8% solution B, 3–81 min is 8–40% phase B, 81–83 min is 40–95% phase B, and 83–90 min is 95% phase B. The peptides were separated and analyzed by DIA MS using a Q-Exactive HF-X mass spectrometer (Thermo Scientific, Waltham, MA, USA).

For DDA mode, the mass spectrometer selected the “top-20” most abundant precursor ions from a full MS spectrum for subsequent MS/MS fragment analysis. The spray voltage was set to 2.1 kV, the funnel RF level at 40, and the heated capillary at 320 °C. Full MS resolutions were set to 60,000, and the full MS AGC target was 3 × A with a maximum inject time (IT) of 30 ms. The mass range was set to 400–1200 *m*/*z*. The AGC target value for fragment spectra was set at 1E5, and the resolution threshold was kept at 30,000 with an IT of 50 ms. The isolation width was set at 1.6 *m*/*z*. The normalized collision energy was set at 28. Only precursors charged between +2 and +7 that achieved a minimum AGC of 8 × 10^3^ were acquired. Dynamic exclusion was set to 40 s to exclude all isotopes in a cluster.

### 4.9. Construction of Fractionated DDA, GPF, and Direct DIA Library

For fractionated DDA sample preparation, 250 μL of 40 biological samples were mixed to generate a pooled urine sample. The EVs isolated from EVrich were lysed, enzymolyzed, and desalted, then redissolved in 20 mM ammonium formate and separated by HPLC of 4 µL/min (U3000, Thermo Xbridge BEH130 C18 column (300 µm × 150 mm, 1.7 µm, Waters)). Phase A is 20 mM ammonium formate, phase B is ACN, 0–14 min is 3% phase B, 14–15 min is 8% phase B, 15–39 min is 29% phase B, 39–43 min is 41% phase B, and 43–60 min is 100% phase B. The fractionation time of the high-performance liquid phase is 60 min in total. In the first 20min, collect one tube of fractionated samples every 1 min, with serial numbers of 1–20, respectively. During the 20–40 min and 40–60 min process, collect the fractionated samples in 1–20 sample tubes in turn. After fractionation, the fractions were dried down using SpeedVac (Jiaimu, Beijing, China). Approximately 8 μL of each reconstituted sample was transferred to a sample vial. The fractionated DDA library was generated using Spectronaut^TM^ software v. 15 (Biognosys, Schlieren, Switzerland). During the generation of the DDA library, the BGS fixed parameters of Spectronaut^TM^ software are used. The enzyme was set to trypsin/P with up to 2 missed cleavages. Carbamidomethylation (C) was selected as a fixed modification, while oxidation (M) and acetylation (protein N-term) were selected as variable modifications. A cut-off of 1% FDR was set at both PSMs and peptides, and a cut-off of 5% FDR at the protein level.

The GPF-DIA library was generated by Spectronaut^TM^ software v. 15 (Biognosys, Schlieren, Switzerland), according to Pino et al. [15]. In brief, we obtained the GPF spectra library (n = 8) from narrow mass ranges of 400–500, 500–600, 600–700, 700–800, 800–900, 900–1000, 1000–1100, and 1100–1200 m/z at the MS1 level. For the MS2 level, 4 m/z DIA spectra were obtained for each MS1 range. The effective isolation window after deconvolution is only 2 m/z. All the DIA data were first searched against the 2021 Human FASTA file with the default setting downloaded from the UniProt website (https://www.uniprot.org, accessed in 22 May 2021). The library search for the GPF-DIA was established using the same parameters as the fractionated DDA spectral library described above.

The MS data were analyzed using the Direct-DIA analysis function of Spectronaut^TM^ software 15 (Biognosys, Schlieren, Switzerland), using the human source database downloaded from the Uniprot website (https://www.uniprot.org, accessed on 22 May 2021) as the database for Direct-DIA analysis, and the search library parameters were the default parameters of the BGS Factory Setting for the Direct-DIA analysis process. The library search for the Direct-DIA was established using the same parameters as the fractionated DDA spectral library described above as well.

### 4.10. Statistical Analysis

The proteomic matrix was z-score transformed in terms of the need for normalization of certain statistical analyses. The statistical power of our study design, calculated in PASS, is 0.81. All statistical analyses were performed using Stata 14.0 (Stata Corp., College Station, TX, USA) or R (version 4.0.5). Descriptive statistics are presented as mean (±SD (standard deviation)) or median (interquartile range) for continuous variables and frequency (percentage, %) for categorical variables.

For more comprehensive analyses, we first performed a statistical difference analysis between the two groups (i.e., prostate cancer cases vs. non-prostate cancer controls) based on the Mann--Whitney test. The differentiated EV proteins found in more than one search strategy were then selected and maintained for further analyses, in which the average abundance was computed and extracted as a proteomic matrix with only identified differentiated EV proteins. The GO (https://www.genome.jp/go/, accessed on 16 August 2022) [43] and DrugBank (https://go.drugbank.com, accessed on 16 August 2022) [44] databases were used to annotate the mechanistic pathways and druggable targets of identified EV proteins. Then, the STRING database v.11.0 (https://string-db.org, accessed on 16 August 2022) [45] was used to retrieve the protein-protein interactions (PPIs) between components of the EV proteomic signature detected in the urine of prostate cancer cases and non-prostate cancer controls. A high confidence (0.9) score was applied. The active interaction sources were experiments and curated databases. Logistic regression analysis was used to evaluate the association between each consistently differentiated EV protein and prostate outcome with adjustments of age, BMI, prostate size, and PSA.

We used a random forest model for accessing the performance of classifying prostate cancer cases and non-prostate cancer controls [46]. Random forest is an ensemble learning method based on the construction of many classification trees. The main benefits of the method are its robustness against overfitting, user-friendliness, and easy interpretation of the model [46]. In the current study, we aimed to apply the random forest-based modeling of clinical information and identified EV proteins to develop a model to classify the outcome of prostate cancer and examine the additional values of EV proteins that contribute to distinguishing prostate cancer and prostatitis/prostatosis. The features included in the analysis were set as (1) 3 conventional clinical factors, including age + prostate size + BMI; (2) age + prostate size + BMI +PSA; and (3) age + prostate size + BMI + PSA + EV protein. The label variable was the prostate outcome. The fitted model is based on the discovery cohort (40 samples) and validated based on the validation cohort (20 samples). We used a 5-fold cross-validation strategy in the discovery cohort (i.e., training dataset) for the determination of the optimal parameters and increase of the classification performance. Then, the established model with the optimal settings was used to make a classification on the validation cohort (i.e., test dataset), where the receiver operating characteristic (ROC) curves were generated by the pROC package in R [47]. *p* values less than 0.05 were considered statistically significant.

## 5. Conclusions

In summary, through a robust platform to isolate EVs and analyze EV proteins, the current study presents an approach to identify molecular markers for disease detection and demonstrates that EVs provide promising sources and values to existing clinical diagnosis for better accuracy and specificity.

## Data Availability

The mass spectrometry raw data files have been deposited at https://www.ebi.ac.uk accessed on 16 October 2022 can be accessed via dataset identifier: PXD037506. Username: reviewer_pxd037506@ebi.ac.uk; Password: mgR28fN1.

## Supplementary Materials

The following supporting information can be downloaded at: https://www.mdpi.com/article/10.3390/molecules27238155/s1, Figure S1: The Number and Abundance Range of EV Proteins based on Different Methods; Figure S2: Association of Three Identified EV Proteins with Prostate Cancer Outcome and PSA; Table S1: Demographic and Clinical Characteristics of Participants in the Discovery Cohort; Table S2: Characterization of Measurement in Different Methods.
