# Supplementary material for: High Throughput Isolation and Data Independent Acquisition Mass Spectrometry (DIA-MS) of Urinary Extracellular Vesicles to Improve Prostate Cancer Diagnosis
Source: Molecules. 2022 Nov 23;27(23):8155. doi: 10.3390/molecules27238155 (PMC9737666; doi:10.3390/molecules27238155)
Supplement: Supplementary file 1 [file molecules-27-08155-s001.zip › molecules-2015773-supplementary.pdf]

Supplementary Table S1 Demographic and Clinical Characteristics of Participants in the Discovery Cohort.

Characteristics	PC	NPC	*P*-value [a]
	***n* = 20**	***n* = 20**	
**Age (year)**	68.60 ± 8.19	66.15 ± 5.56	0.275
**BMI (kg/m^2)**	24.98 ± 3.62	24.37 ± 2.40	0.534
**Prostate Size (mm^3)**	77.00 ± 34.04	87.50 ± 44.39	0.407
**PSA (ng/ml)**	25.98 ± 20.77	8.28 ± 3.61	0.006

[a] Calculated by Mann-Whitney test between prostate cancer cases and non-prostate cancer controls.

Abbreviation: PC, prostate cancer; NPC, non-prostate cancer; BMI, body mass index; PSA, prostate-specific antigen.

P-value <0.05 was considered statistically significant.

Supplementary Table S2. Characterization of Measurement in Different Methods.

**Method**	**Precursor Range (m/z)**	**Minimal Localization Threshold**	**The Number of EV Protein**	**The Number of EV Peptides**
**Search Library**				
**DDA Library**	400-1200	/	**6,104**	**40,955**
**GPF Library**	400-1200	/	**3,535**	**28,522**
**Discovery Cohort**				
**Direct-DIA**	400-1200	0.75	**2,969**	**30,782**
**GPF-DIA**	400-1200	0.75	**3,282**	**27,668**
**DDA-DIA**	400-1200	0.75	**3,273**	**22,690**
**Validation Cohort**				
**Direct-DIA**	400-1200	0.75	**2,923**	**29,911**

A urine EV sample was used to compare Direct-DIA (a direct searching based on Uniprot library was conducted, without a requirement of spectral libraries to analyze DIA data, which was denoted as Direct-DIA), GPF-DIA (a gas-phase fractionated (GPF) library-based searching was denoted as GPF-DIA) and DDA-DIA (a fractional DDA based searching was denoted as DDA-DIA), with 0.75 minimal localization thresholds. The direct DIA, GPF DIA and DDA-DIA were performed at a precursor range: 400-1200 m/z.

Abbreviation: EV, extracellular vesicles; DDA, data dependent analysis; GPF, gas-phase fractionated; DIA, data independent analysis.

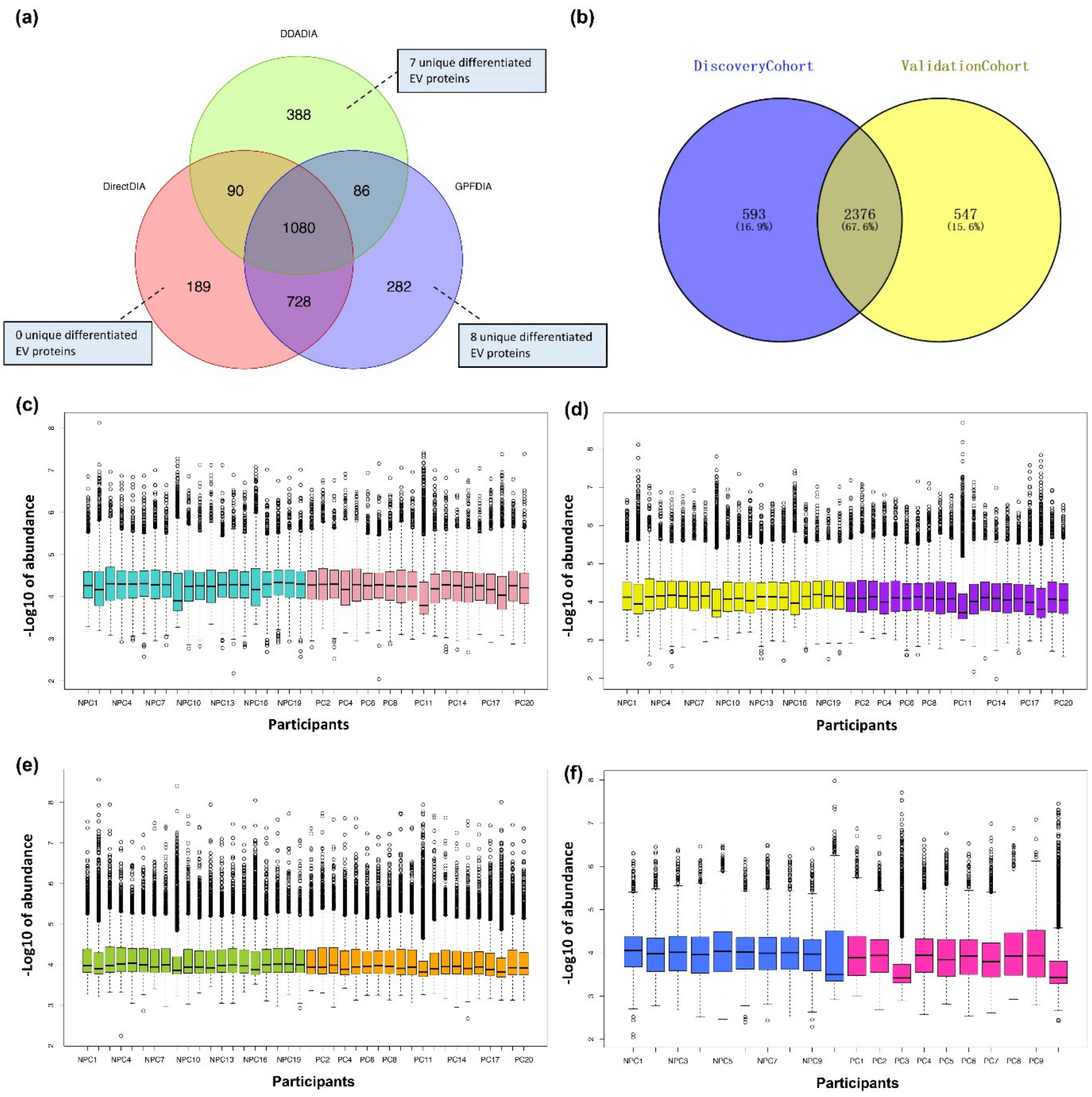

Supplementary Figure S1 The Number and Abundance Range of EV Proteins based on Different Methods. (a) the overlap of urine EV proteins among three methods. (b) the overlap of urine EV proteins between discovery cohort and validation cohort. (c) range of abundance for each participant included in the discovery cohort based on Direct-DIA. (d) range of abundance for each participant included in the discovery cohort based on GPF-DIA. (e) range of abundance for each participant included in the discovery cohort based on DDA-DIA. (f) range of abundance for each participant included in the validation cohort based on Direct-DIA. **Abbreviations:** EV, extracellular viscles; DIA, data independent acquisition; DDA, data dependent analysis; GPF, gas-phase fractionated; PC, prostate cancer; NPC, non-prostate cancer.

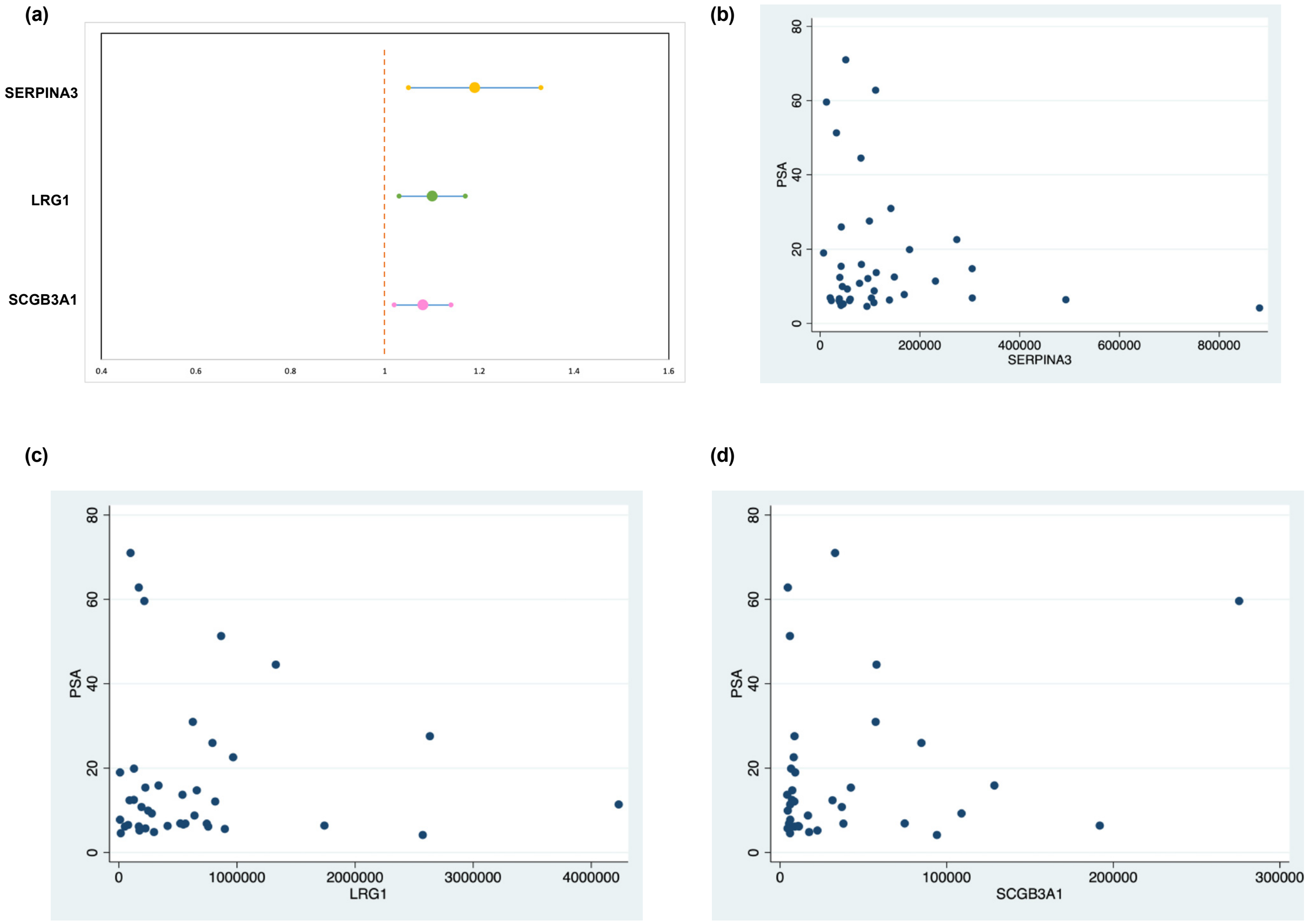

Supplementary Figure S2 Association of Three Identified EV Proteins with Prostate Cancer Outcome and PSA. (a) association between identified EV proteins and prostate cancer outcome. (b)-(d) scatter plot between each identified EV protein (SERPINA3, LRG1 and SCGB3A1, respectively) and PSA.

Abbreviations: EV, extracellular vesicles; PSA, prostate-specific antigen; SERPINA3, Alpha-1-antichymotrypsin, LRG1, Leucine-rich alpha-2-glycoprotein; SCGB3A1, Secretoglobin family 3A member 1.
